# Unveiling Neonatal Appendicitis: A Unique Case of Chronic Appendicitis With Fibrosis and Cystic Mass Presentation

**DOI:** 10.7759/cureus.94060

**Published:** 2025-10-07

**Authors:** Antonio Santana Veliz, Kate Van Loveren, Shilpa Reddy, Daniel Zaccarini, Ravikumar Hanumaiah

**Affiliations:** 1 Diagnostic Radiology, State University of New York Upstate Medical University, Syracuse, USA; 2 Pediatric Surgery, State University of New York Upstate Medical University, Syracuse, USA; 3 Pathology, State University of New York Upstate Medical University, Syracuse, USA; 4 Radiology, State University of New York Upstate Medical University, Syracuse, USA

**Keywords:** abdominal cystic mass, appendiceal abscess, neonatal appendicitis, neonatal surgery pediatric gi surgery, prenatal appendiceal perforation

## Abstract

Neonatal appendicitis is a rare condition, with limited comparable case reports and studies available in the literature. Clinical presentation can be variable, but typically includes abdominal distension, fever, decreased feeding, and vomiting.

This case study describes a previously healthy 21-day-old female who presented to the emergency department with a one-day history of abdominal distension and decreased feeding. Imaging, including abdominal X-ray, ultrasound, and MRI, demonstrated a cystic mass in the right lower quadrant of unclear etiology. Exploratory laparotomy revealed a gelatinous, bilobed cystic-appearing mass located deep to a loop of small bowel in the right lower quadrant, necessitating an ileocecal resection.

Histopathology demonstrated foci of acute inflammation with abscess formation, necrosis, and a surrounding exuberant fibroblastic reaction.

This case contributes to the limited understanding of neonatal appendicitis and offers valuable insight into its presentation, imaging findings, and surgical management.

## Introduction

Appendicitis is characterized by inflammation of the appendix and is a rare occurrence in the neonatal period. It occurs in fewer than 2% of infants less than two years old. The incidence of this disease is 0.04-0.2% of neonates, affecting male more than female infants in a ratio of 3:1. Classic appendicitis is the inflammation of the appendix, typically caused by obstruction of its lumen, leading to bacterial overgrowth. The non-specific and varied presentation of this patient population makes pre-operative diagnosis difficult. The most common symptoms include abdominal distention, fever, decreased feeding or refusal to feed, and vomiting [1]. There are some studies lending to the value of ultrasound (US) in establishing a pre-operative diagnosis; however, most cases are identified operatively with support from histological analysis [1].

## Case presentation

**Figure 1:**
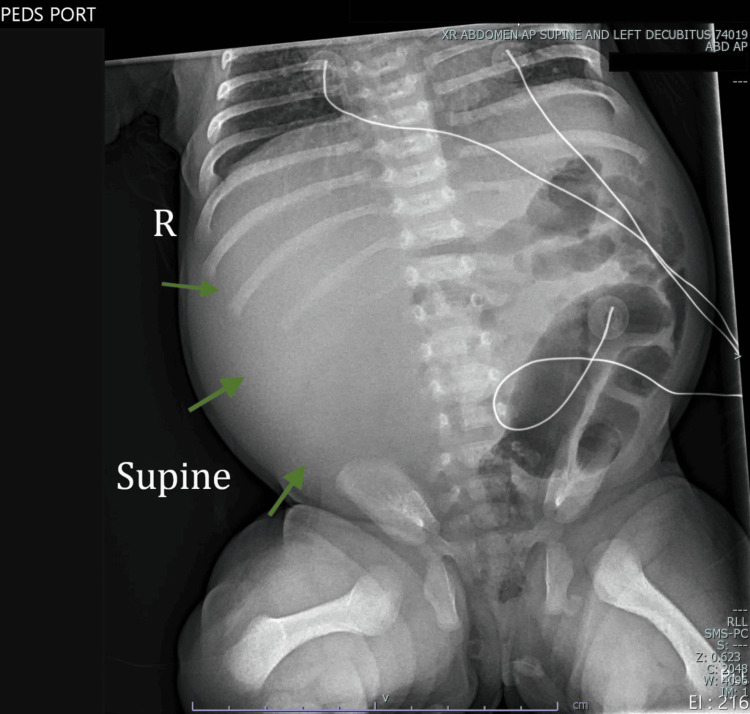
Marked paucity of gas within the small bowel, with displacement of bowel loops into the left flank. These findings are concerning for a large right flank mass lesion displacing the bowel loops leftward.

**Figure 2:**
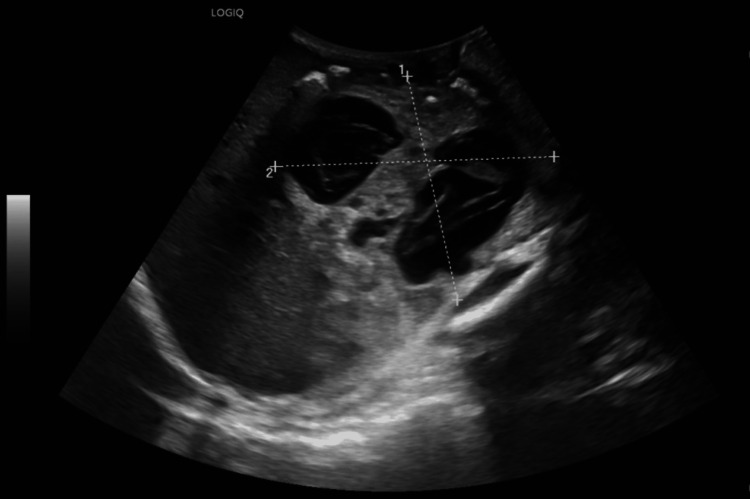
Large complex cystic mass with internal septations arising from the right retroperitoneum.

**Figure 3:**
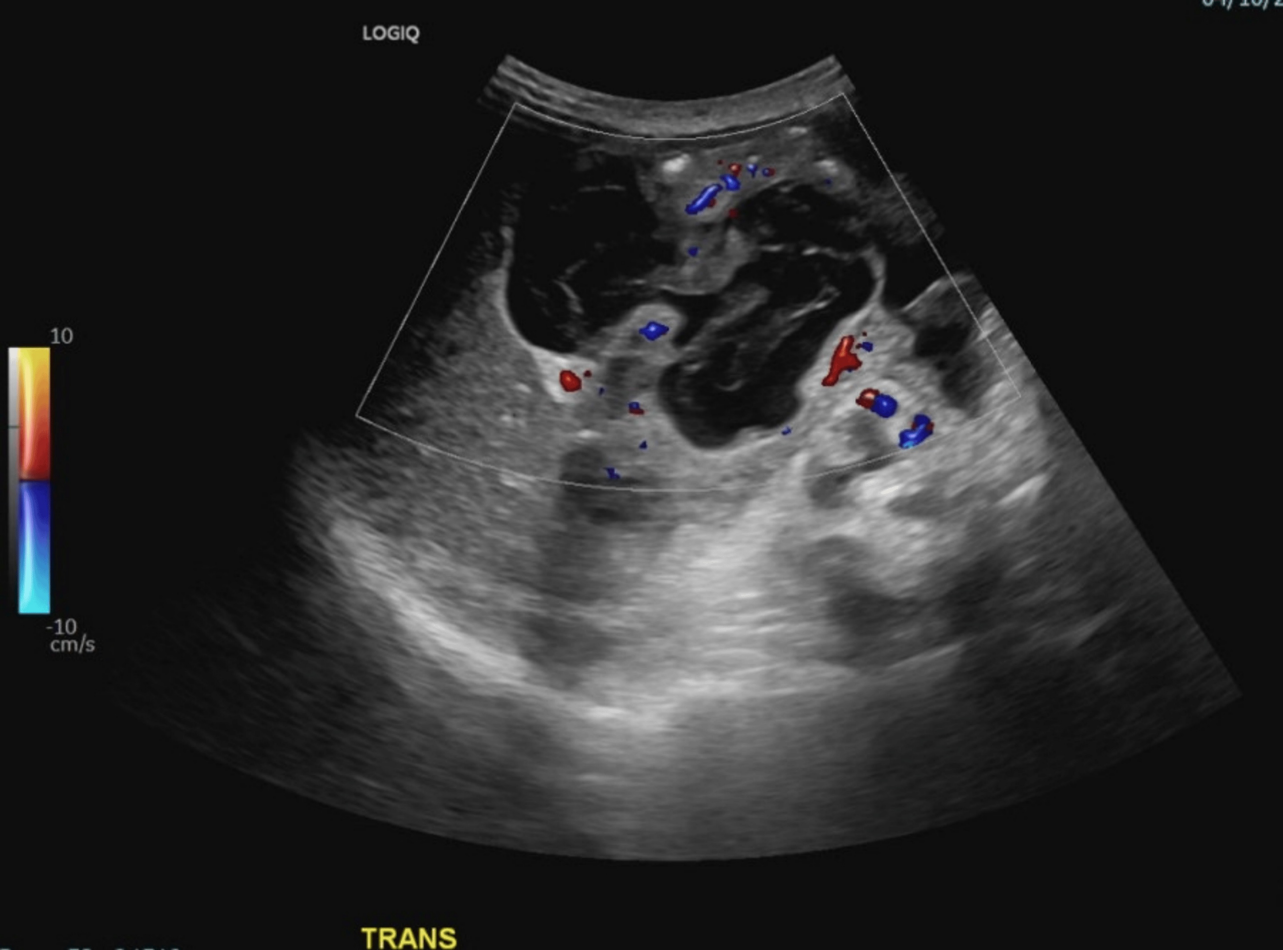
Large complex cystic mass with vascularity arising from the right retroperitoneum.

**Figure 4:**
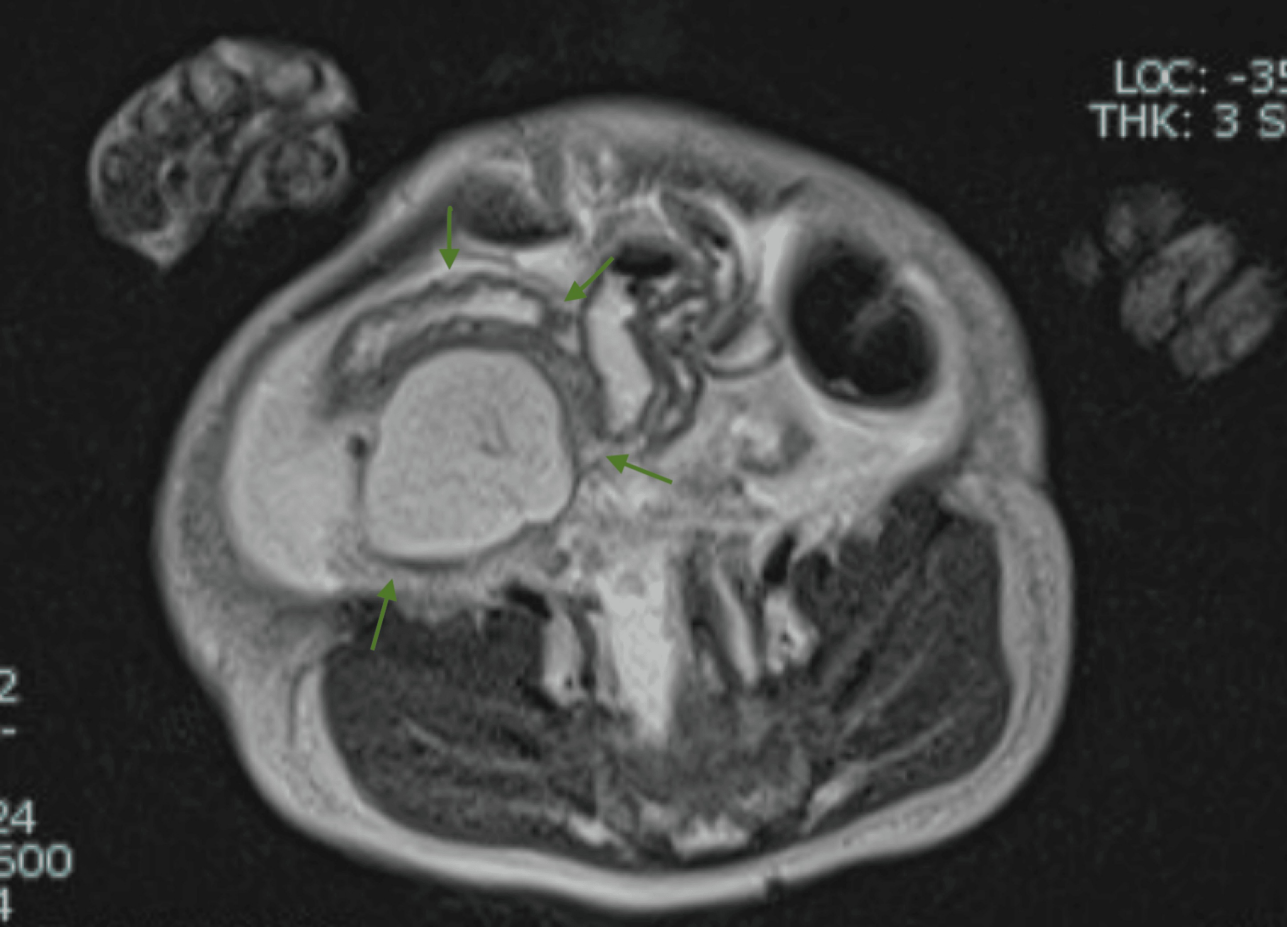
Axial T2 half-Fourier acquisition single-shot turbo spin echo (HASTE) image demonstrates a large cystic mass in the right upper abdomen with moderate ascites, displacing bowel loops without signs of obstruction and with no clear organ of origin.

**Figure 5:**
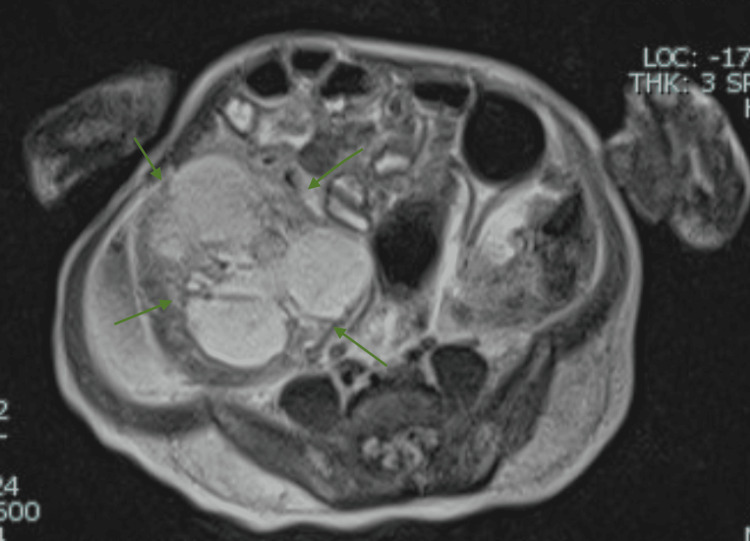
Axial T2 half-Fourier acquisition single-shot turbo spin echo (HASTE) image at the level of the right lower quadrant shows the inferior extent of the same cystic lesion, further confirming its large size and lack of a clear organ of origin.

A 21-day-old female neonate with no significant past medical history presented with a one-day history of abdominal distention, decreased feeding, and increased work of breathing. Initial imaging was obtained with an abdominal AP supine radiograph showing bowel displacement to the left (Figure 1). Abdominal US complete showed a large complex cystic mass measuring 7.5 by 5.4 cm (AP, TR dimension) arising from right retroperitoneum with internal septation and vascularity on color Doppler (Figures 2, 3). Abdominal MRI showed the cystic mass was not associated with any abdominal organs (Figures 4, 5). Based on the imaging, there was an unclear etiology to this cystic mass with differential diagnoses including a cystic teratoma, sarcoma, mesenteric neoplasm, or possible right ovarian mass. Imaging, specifically MRI, however, was able to rule out signs of metastatic disease, intussusception, and malrotation. It was decided that an exploratory laparotomy was necessary for further evaluation

During the pre-operative course, the patient was experiencing intermittent fevers and was found to be positive for parainfluenza virus. A sepsis workup was negative as blood and urine cultures did not result with any evidence of microorganism growth. As a precaution, the patient was placed on ampicillin and ceftriaxone for three days.

**Figure 6:**
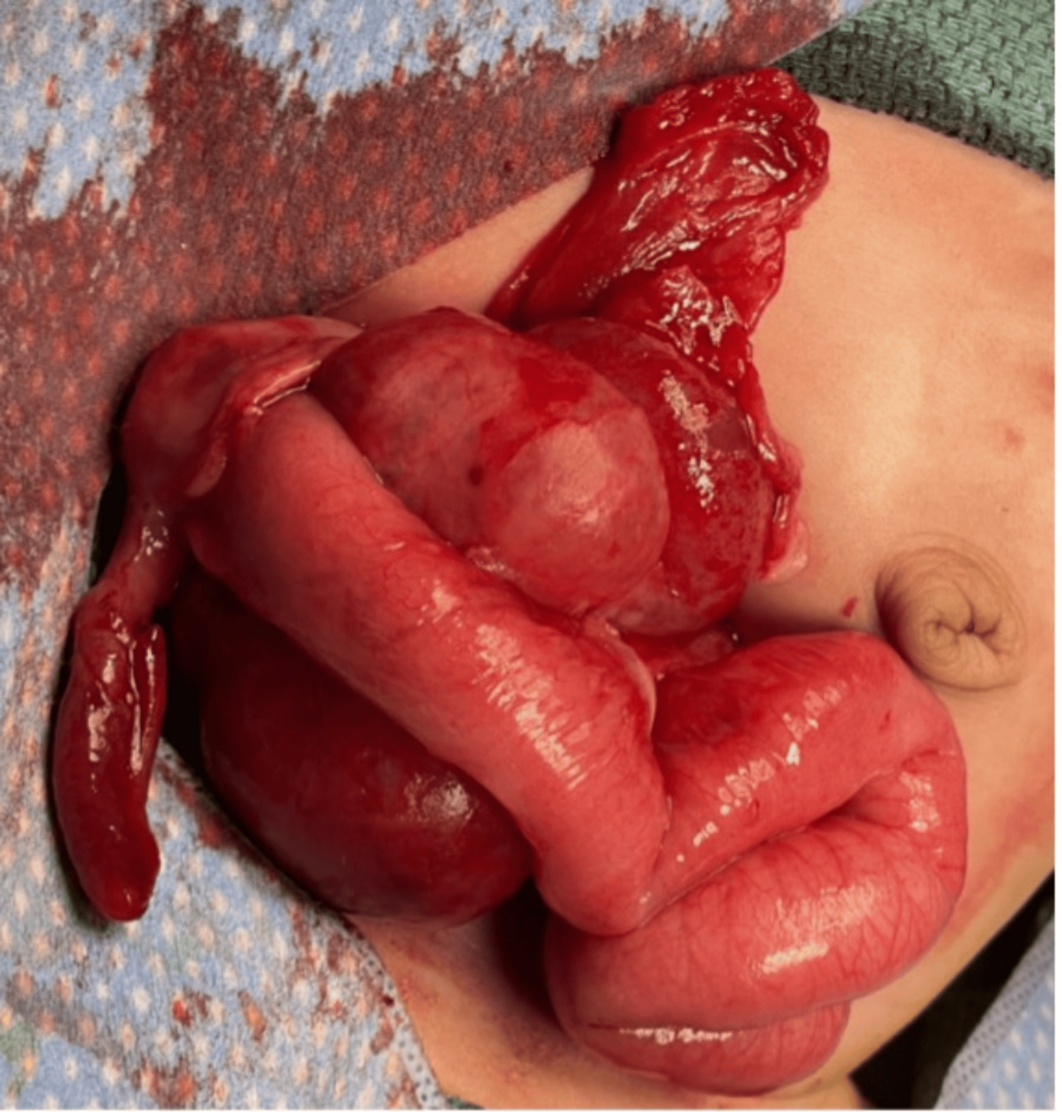
Intraoperative image shows a bilobed, gelatinous, cystic-appearing mass adherent to the ileocecal region and closely associated with the cecum.

Intraoperatively, there were ascites upon entering the peritoneum, for which a sample was collected. On further exploration of the right lower quadrant deep to the loop of the small bowel, a gelatinous, cystic appearing, bilobed mass was identified (Figure 6). On palpation, the mass felt fixed to the retroperitoneum and the overlying bowel. It bordered the cecum, ascending colon, root of the small bowel mesentery, and ileocecal region. An ileocecectomy was performed to remove the mass and associated attached bowel segment.

**Figure 7:**
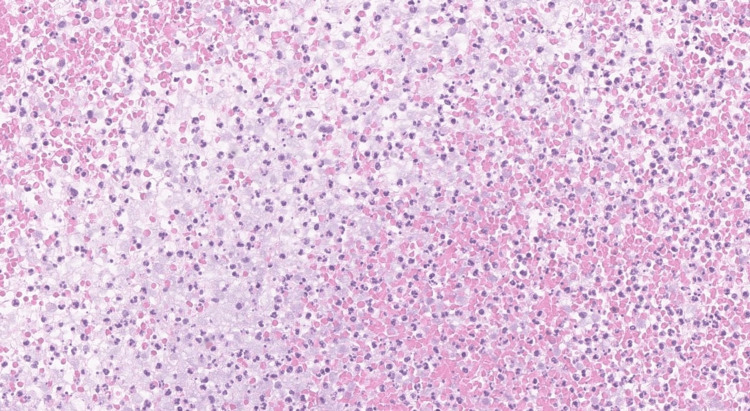
Fragments of abscess cavity containing acute inflammatory infiltrate with abundant neutrophils and necrotic debris (H&E, 20x).

**Figure 8:**
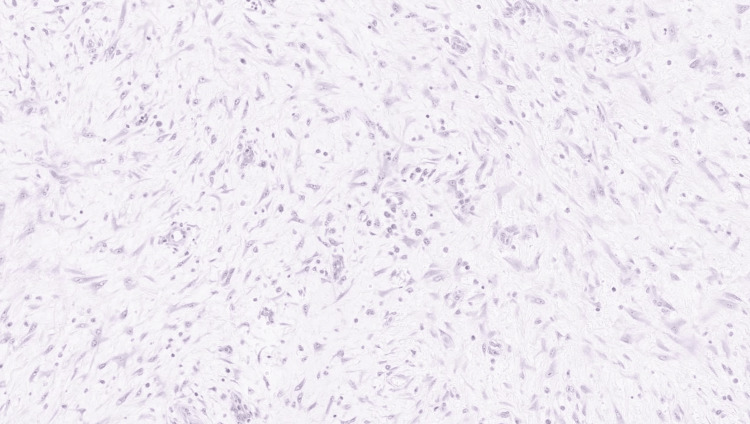
Spindle-shaped myofibroblast proliferation with no evidence of malignancy (H&E, 10x).

The pathology showed the presence of reactive mesothelial cells and polymorphous lymphocytes which were mostly histiocytes (Figure 7). The bowel specimen included sections of the ileum, ileocecal valve, appendix, colon, and associated lymph nodes. In the ileum and cecum, there was acute inflammation with abscess formation, necrosis, and overlying fibroblastic reaction, consistent with chronic inflammation (Figure 8). The appendix showed serosal adhesions and acute serositis. Lymph nodes displayed no pathology. There was no evidence of malignant features in the preliminary pathology review.

Post-operatively, the patient received cefazolin for three days prophylactically and acetaminophen for pain management. Mild peri-orbital edema and redness around the abdominal incision were noted, likely related to intra- and post-operative fluid administration. By day three, the patient had regular bowel movements, tolerated oral feedings well, and showed improvement in incision erythema, indicating return to baseline. The patient was discharged after a six-day hospital stay.

After discharge from the hospital, 10 days later, the patient was reported to have residual abdominal distention, was easily passing gas and having bowel movements, and oral feedings were normal. Approximately 50 days later, the patient was doing well with no complications. At the three-month follow-up appointment, on evaluation with US the patient had no abdominal mass, though there was limitation to the study due to bowel gas. The patient will follow up in one year with a US study.

## Discussion

Neonatal appendicitis (NA) is a rare finding with severe risk of complications including sepsis and perforation.

For the presentation of NA, patients typically exhibit non-specific gastrointestinal and systemic symptoms. Schwartz et al. reported that among 32 cases, the most common findings included abdominal distention in 75% of cases, along with vomiting, refusal to feed, and temperature instability. In a separate study of 69 neonates with NA, the most frequently reported symptoms were abdominal distention (52.2%), fever (27.5%), decreased feeding or refusal to feed (23.2%), and vomiting (21.7%) [2]. These reviews indicate that the clinical presentation of NA often involves general gastrointestinal complaints, with no consistent pattern of signs or symptoms specific to the disease process [2].

Most studies indicate the rarity of this disease can be due to the shape of the fetal appendix (funnel-shaped with a wide base towards the opening of the cecal junction), obstructive causes such as colonic atresia, ischemia from vascular insufficiency, or infectious processes including necrotizing enterocolitis [3]. Though the cases are rare, the prognosis can be severe with complications including perforation and sepsis that require immediate intervention. Given that most cases are identified operatively, it is necessary to expand on the literature to identify more consolidated understandings of the presentation, diagnostic findings, and management for this disease.

Pre-operatively, US has shown to be a potentially successful modality for identifying NA. In a study of neonates 10-17 days old, US was used to diagnose NA with findings that included ileocecal bowel dilation and thickening, right lower quadrant effusion, and abscess formation [3].

In a review of 69 cases of NA, 65 received a pre-operative US with findings that showed outer wall thickness, luminal fluid, blood flow, and right lower quadrant fluid collection [3]. These studies indicate that the US can be a beneficial tool to help diagnose NA; however, due to the small number of NA cases it is difficult to establish an official consensus of a pattern of findings that best point to NA.

Case studies have shown a variety of intraoperative and histological findings in NA. Some cases report the presence of ascites, a calcified mass with dense adhesions, or a gangrenous-appearing appendix with surrounding adhesions. In one instance, histology revealed mucosal lymphomononuclear infiltration and organizing serositis with perforation, while another case showed dense fibrinosuppurative infiltrates and transmural granulation within the appendiceal lumen [4].

The literature presents a wide range of potential presentations for NA, with no clear diagnostic criteria, making it difficult to determine whether our case fits with the typical presentation, represents a rare variation of NA, or suggests an entirely different diagnosis. In our case, the presentation mirrored the most commonly reported symptoms of NA, including abdominal distension and reduced feeding. However, ultrasound revealed a cystic mass, which is unusual since prior literature on NA doesn’t typically show cystic masses on imaging. This could be attributed to the limited number of cases available or the unusual nature of this particular case. Histopathological analysis of the surgical specimen showed signs of an inflammatory process with necrosis affecting the ileum, cecum, and appendix. The appendix showed adhesions, similar to other cases. However, unlike most cases, the pathology revealed a fibroblastic reaction in the affected area.

The presence of fibrosis suggests the possibility of long-standing appendicitis, potentially starting very early, either in utero or shortly after birth. While prenatal appendicitis is rare, the few cases that have been reported typically present acutely within a few days after birth and require surgical intervention. In these cases, histology does not show signs of fibrosis. In contrast, chronic appendicitis in older children is often associated with both inflammation and fibrosis. Based on these observations, we hypothesize that our case could represent a form of chronic neonatal appendicitis with an unclear onset, potentially starting in utero or soon after birth.

## Conclusions

Given the small volume of research on this disease, there is little insight into the characteristic presentation, US findings, and histological analysis of NA. This report demonstrates a unique case of chronic NA with findings of fibrosis, as a consequence of possible antenatal or early neonatal appendicitis.
